# Supplementary material for: How norm violators rise and fall in the eyes of others: The role of sanctions
Source: PLoS One. 2021 Jul 29;16(7):e0254574. doi: 10.1371/journal.pone.0254574 (PMC8324048; doi:10.1371/journal.pone.0254574)
Supplement: S1 File — (PDF) [file pone.0254574.s001.pdf]

Supplemental material

Data collection, confirmatory & exploratory findings, and data sharing

The data for Study 1 were collected by the 4th author, together with the 2nd and last author. At that time, the 4th author predicted (among others) mediation from norm violation to power via volition, and expected that power inferences would be reduced by sanctions. The 4th author included several additional variables that were not of central interest to the 1st author when they continued the project with Study 2 (together with the 2nd, 3rd, and last author). These variables are therefore not reported in the main text, but are described in the file CodebookS1.xlsx. Likewise, we measured additional variables in Study 2 that are not reported in the main text. These are described in CodebookS2.xlsx. Both files can be accessed from https://osf.io/xjpe5/.

We share the complete datasets including the variables that were not reported in the text (likewise accessible from https://osf.io/xjpe5/. We denote which variables this pertains to in the codebooks.

Full text for the scenario in Study 1

English translation of the German stimulus material.

A traveler enters a local train station.

- Rule abidance: In the building, he walks by a convenience store without buying anything, and goes to a ticket machine, where he buys a ticket for a regional train that costs about EUR 5.
- Rule violation: In the building, he walks by a ticket machine without buying a ticket, and goes to a convenience store, where he buys a coffee and some snacks that cost about EUR 5.

Then he goes to the track his train leaves from. Just as the train pulls into the station, he pulls his phone out of his pocket to read a message that he had just received. Reading the message, he boards the train and sits in a window seat.

After a while, the train conductor enters the carriage and asks the passengers to please show their tickets if they had just boarded the train. The conductor walks down the aisle and checks each

newly boarded passenger. When he stands next to the traveler, he also asks him to show his ticket.

- Rule abidance: The traveler looks for his ticket in his pockets and cannot find it. He looks up and tells the conductor candidly that he bought a ticket but cannot find it anymore.
- Rule violation: The traveler looks up and lies to the conductor as he tells him that he bought a ticket and that his ticket had already been checked.

The conductor frowns as he looks at the traveler.

- No sanction:
  - Rule abidance: The conductor tells the traveler that this is not a problem and the will make an exception for the traveler. Usually, passengers who cannot show valid tickets have to pay a fine of EUR 40 in addition to having to pay for a new ticket. Then the conductor moves on to the passenger sitting in front of the traveler.
  - Rule violation: Then the conductor moves on to the passenger sitting in front of the traveler.
- Sanction:
  - Rule abidance: The conductor tells the traveler that this is a problem and he cannot make an exception for the traveler. Passengers who cannot show valid tickets have to pay a fine of EUR 40 in addition to having to pay for a new ticket. The conductor collects the fine from the traveler and issues him a new ticket. Then the conductor moves on to the passenger sitting in front of the traveler.
  - Rule violation: The conductor insists on seeing the traveler’s ticket. When he cannot show a valid ticket, the conductor tells the traveler that this is a problem and he cannot make an exception for the traveler. Passengers who cannot show valid tickets have to pay a fine of EUR 40 in addition to having to pay for a new ticket. The conductor collects the fine from the traveler and issues him a new ticket. Then the conductor moves on to the passenger sitting in front of the traveler.

Full text for the scenario in Study 2

English translation of the Dutch Stimulus Material.

A traveler enters a local train station.

- Control: In the building, he walks by a convenience store without buying anything, and goes to a ticket machine, where he buys a ticket for a regional train that costs about EUR 5.
- Rule violation (sanctioned and non-sanctioned): In the building, he walks by a ticket machine without buying a ticket, and goes to a convenience store, where he buys a coffee and some snacks that cost about EUR 5.

Then he goes to the track his train leaves from. Just as the train pulls into the station, he pulls his phone out of his pocket to read a message that he had just received. Reading the message, he boards the train and sits in a window seat.

After a while, the train conductor enters the carriage and asks the passengers to please show their tickets if they had just boarded the train. The conductor walks down the aisle and checks each newly boarded passenger. When he stands next to the traveler, he also asks him to show his ticket.

- Control: The traveler looks for his ticket in his pockets and hands it to the conductor.
- Rule violation (sanctioned and non-sanctioned): The traveler looks up and lies to the conductor as he tells him that he bought a ticket and that his ticket had already been checked.

The conductor frowns as he looks at the traveler.

- Control: Then the conductor moves on to the passenger sitting in front of the traveler.
- Rule violation (non-sanctioned): Then the conductor moves on to the passenger sitting in front of the traveler.
- Rule violation (sanctioned): The conductors insists on seeing the traveler's ticket. When he cannot show a valid ticket, the conductor tells the traveler that this is a problem and he cannot make an exception for the traveler. Passengers who cannot show valid tickets have to pay a fine of EUR 40 in addition to having to pay for a new ticket. The conductor

collects the fine from the traveler and issues him a new ticket. Then the conductor moves on to the passenger sitting in front of the traveler.

Full text for sample items in Study 1 and 2

English versions of the German (Study 1) and Dutch (Study 2) scales for which only sample items are presented in the main text. Reverse coded items are denoted with [r].

Perceived Volition (Magee, 2009):

To what extent …

1. are this person's decisions the product of his own will?
2. does this person feel free to do what he wants
3. is this person's behavior a product of his own volition?
4. is this person's behavior involuntary? [r]
5. does this person lack freedom in deciding what to do? [r]
6. is this person's behavior driven by the wishes of other people? [r]

Perceived Power (Anderson & Galinsky, 2006):

1. He can get people to listen to what he says.
2. His wishes do not carry much weight. [r]
3. He can get others to do what he wants.
4. Even if he voices them, his views have little sway. [r]
5. He thinks he has a great deal of power.
6. His ideas and opinions are often ignored. [r]
7. Even when he tries, he is not able to get his way. [r]
8. If he wants to, he gets to make the decisions.

Code book

Study 1

- id: participant number
- gender: "female" vs. "male"
- age: divided into lower (1), middle (2), and upper (3) percentile to ensure anonymity

- violation: experimental condition: “violate” vs. “abide”
- sanction: experimental condition: “no sanction” vs. “sanction”
- volition_1 through 6: corresponds to the volition items reported in the previous section
- power_1 through 8: corresponds to the power items reported in the previous section
- mc.violation_1 and 2: corresponds to the norm violation checks reported in the main text
- mc.sanction_1 and 2: corresponds to the sanction checks reported in the main text
- exclusion: reason for exclusion
- separator: column separating data reported in the main text from additional variables [(1)]

Study 2

- id: participant number
- gender: “female” vs. “male” (a third, non-binary, option was included in this study, but not selected by any participant)
- age: divided into lower (1), middle (2), and upper (3) percentile
- condition: experimental condition: “control” vs. “no sanction” vs. “sanction”
- volition_1 through 6: corresponds to the volition items reported in the previous section
- power_1 through 8: corresponds to the power items reported in the previous section
- mc.violation_1 through 3: corresponds to the norm violation checks reported in the main text
- mc.sanction_1 through 3: corresponds to the sanctioning items reported in the main text
- exclusion: reason for exclusion
- separator: column separating data reported in the main text from additional variables [(1)]

[(1)] See the files “codebookS1.xlsx” and “codebookS2.xlsx” for a description of all variables.

Test of moderated mediation in Study 1

In this section we provide a more detailed explanation for the moderated mediation test as conducted in Study 1. To this purpose, we first explain how our moderated mediation model resembles a simple mediation model, before detailing how a potential moderating effect of

sanctions can be tested. Our explanations are largely based on Hayes (2013, 2015).

Figure S1. Simple Mediation Model (Left), and Conceptual (Center) and Statistical (Right) Models of the Proposed Moderating Effect of Sanctions in Study 1.

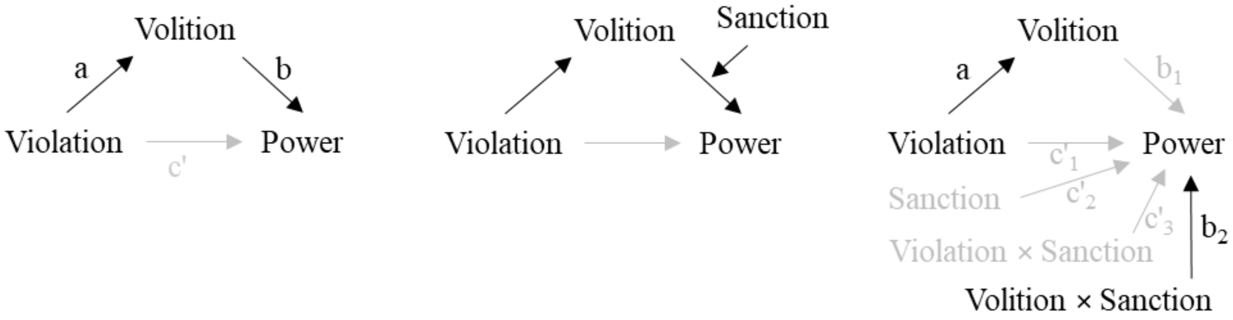

A simple mediation model as in the left panel of Figure S1 encompass a direct effect (c') and an indirect effect that consists of the product of an "a path" (the effect of the predictor on the mediator) and a "b path" (the effect of the mediator on the outcome variable). In this case, the indirect effect is simply ab. We can represent our moderated mediation model in a similar way. The conceptual model (Figure S1 center) can be translated into a statistical model (right), which is captured by the following two equations:

(1) $\text{volition} = i_{\text{volition}} + a\ \text{violation} + e_{\text{volition}}$

(2) $\text{power} = i_{\text{power}} + b_1\ \text{volition} + b_2\ \text{volition} \times \text{sanction} + \ldots + e_{\text{power}}$

where i denotes intercepts, e error terms, and ellipses represent direct effects (c'_1 violation + c'_2 sanction + c'_3 volition × sanction) which are not of interest here.[1] Ignoring intercepts, errors, and direct effects for simplicity, the two equations can be rewritten by substituting (1) into (2) and refactoring:

(3) $\text{power} = b_1\ (a\ \text{violation}) + b_2\ (a\ \text{violation}) \times \text{sanction}$

(4) $\text{power} = (\underline{a \times (b1 + b2\ \text{sanction})})\ \text{violation}$

Equation (4) shows that – just like for a simple mediation model – the indirect effect in our

[1] The c_3 path in the statistical model is not apparent from the conceptual model. However, Hayes (2013) recommends to always include interaction terms for both direct and indirect effects even when only the interaction term for the indirect effect is of interest. If the interaction term involving the direct effect is non-significant, this will show; on the other hand, failing to model an existing interaction term will result in a misspecified model. As a consequence, one should always include the term, Hayes argues.

moderated mediation model (underlined) consists of the product of an “a path” (a) and a “b path” (the sum of b_1 and b_2 conditional on the presence vs. absence of sanctions).

After having demonstrated that our moderated mediation model is not much different from a simple mediation model, and thereby illustrating how an indirect effect can be calculated, we next explain how a (potential) moderating effect of sanctions can be tested. To this purpose, we rewrite equation (4) to isolate the role of sanctions (underlined):

(5) $\text{power} = (ab_1 + \underline{ab_2}\ \text{sanction})\ \text{violation}$

In the absence of sanctions (sanction = 0), the indirect effect will simply be ab_1. In the presence of sanctions (sanction = 1), the indirect effect will become $ab_1 + ab_2$. Sanctions moderate the indirect effect if the two indirect effects differ. As both indirect effects encompass ab_1, the two indirect effects will differ when ab_2 is significant. The product ab_2 therefore provides a test of moderated mediation.

References

Anderson, C., & Galinsky, A. D. (2006). Power, optimism, and risk-taking. *European Journal of Social Psychology*, *36*, 511–536.

Hayes, A. F. (2013). *Introduction to mediation, moderation, and conditional process analysis. A regression-based approach*. *New York, NY: Guilford*. New York, NY: Guilford Press. doi:978-1-60918-230-4

Hayes, A. F. (2015). An index and test of linear moderated mediation. *Multivariate Behavioral Research*, *50*. doi:10.1080/00273171.2014.962683

Magee, J. C. (2009). Seeing power in action: The roles of deliberation, implementation, and action in inferences of power. *Journal of Experimental Social Psychology*, *45*, 1–14.
