# Supplementary material for: How norm violators rise and fall in the eyes of others: The role of sanctions
Source: PLoS One. 2021 Jul 29;16(7):e0254574. doi: 10.1371/journal.pone.0254574 (PMC8324048; doi:10.1371/journal.pone.0254574)
Supplement: Attachment — Submitted filename: Response to reviewers.docx [file pone.0254574.s002.docx]

**Responses to Editor comments**

1. Please ensure that your manuscript meets PLOS ONE's style requirements, including those for file naming.

**Response:** We have carefully checked that our manuscript meets PLOS ONE's style requirements.

2. During our internal checks, the in-house editorial staff noted that you conducted research or obtained samples in another country (for study 1). Please check the relevant national regulations and laws applying to foreign researchers and state whether you obtained the required permits and approvals. Please address this in your ethics statement in both the manuscript and submission information.

**Response:** The study was approved by the Ethical review board of the Faculty of Social and Behavioral Sciences at the University of Amsterdam, but data was collected online from a convenience sample of participants in Germany. The Code of Conduct of the German Psychological Society stipulates ethical considerations (https://www.dgps.de/index.php?id=85) for research with human participants, which do not reference special permits required for international researchers. Further, ethical considerations in research in Germany are subject to the same codified ethical guidelines as in the Netherlands, namely the Helsinki Declaration and European data protection regulations. Therefore, no additional permits were required to conduct this research. We have added this information in the ethics statement in both the manuscript (Methods section Study 1) and submission.

3. Thank you for your ethics statement:

'Institutional review board: Ethical review board, Faculty of Social and Behavioral Sciences, University of Amsterdam, Approval numbers: 2014-WOP-3498 and 2017-COP-8050
Consent obtained written online anonymously'

a. Please amend your current ethics statement to confirm that your named institutional review board or ethics committee specifically approved this study.

b. Once you have amended this/these statement(s) in the Methods section of the manuscript, please add the same text to the “Ethics Statement” field of the submission form (via “Edit Submission”).

**Response:** We have amended the ethics statements in the Methods sections of Study 1 (p. 6) and Study 2 (p. 14), respectively and we have added the same text to the “Ethics Statement” field of the submission form.

**Responses to Reviewer #1:**

The paper tackles an interesting and important topic, it is well written and all analyses are described clearly, including limitations and indicating which aspects where exploratory or previously hypothesized.
I thus have only a few minor comments:

**Response:** Thank you for the overall positive evaluation of our work and your constructive comments.

1. the authors remain a bit silent about which online participant pools they use. Is this something comparable to MTurk, or are these student participants?

**Response:** The online participant pool (*M_age_=*23.78) was recruited from passers-by at a German university campus as well as through social media. We have added this information on page 6.

2. Some of the arguments for the difference in effects between the first study and previous studies are based on arguments about the German Railway norms, however, the second study then is (again, as previous studies, I guess) done in the Netherlands. It would be interesting to understand whether this - as opposed to the design differences - plays a role for the effects, whether norms and norm abidance are perceived differently in the two systems.

**Response:** There are cultural differences as well as similarities between the two countries on dimensions that are relevant to norm violation. Germany scores higher than the Netherlands on cultural tightness, which relates to the importance that is attached to rules and the severity of punishment for violations (Gelfand et al., 2011). In Study 1 (German sample) we found that non-sanctioned norm abiders were perceived to have violated norms to a greater extent than sanctioned norm abiders, although the effect size was small. We could speculate that participants were aware of the railway operators’ right to fine the traveler and – due to their tightness - were indignant that the traveler got away without this fine.

Germany and the Netherlands are comparable in terms of individualism, the degree to which uniqueness, personal achievement, and self-expression are valued (as opposed to group harmony and collective outcomes). A cross-cultural comparison of responses to norm violations (including data from Germany and the Netherlands) revealed that particularly respondents from individualistic cultures (including Germany and the Netherlands) perceive norm violators as more powerful than norm abiders (Stamkou et al., 2019).

Finally, regarding the specific scenario in this study, we would like to note that Germany and the Netherlands are European Schengen states, allowing free traveling among these states, and therefore use similar terms and conditions for train travel. In the discussion section, we now address the possibility of cultural differences as a subject for future research.

3. the authors themselves discuss that one problem with the results is the hypothetical character. There are by now several studies from the team of Marie-Claire Villeval (Lyon) which use real settings to study similar questions. I am wondering whether the authors are aware of this research and whether they might consider doing more real world studies in the future.

**Response:** The work by Villeval and her colleagues (e.g., Dai et al., 2018) is very interesting and employs creative methods. Importantly, this work is mainly focused on the intrapersonal drivers of norm breaking behaviors (e.g., what determines cheating behavior in individuals) and not on interpersonal dynamics (i.e., how do others respond to observing individuals who violate the norms), which is the approach we take in our work.

Responses to norm violations can indeed be studied in various ways, which entail different trade-offs between ecological validity and experimental control. We have used a variety of approaches in our work, including scenarios, pictures, video clips, recalled situations, and live interactions with trained actors to investigate responses to norm violations (Stamkou et al., 2016, 2018, 2019; Van Kleef et al., 2011, 2012). Scenarios, pictures, and video clips afford greater experimental control, whereas recalled situations and live interactions afford greater ecological validity. Which method is most suitable in a given study depends on the nature of the research question in combination with the possibilities and constraints of the different methodological approaches. For the current project, we prioritized experimental control to enable causal conclusions about the effects of sanctioning on responses to norm violators. Although we acknowledge the limitations of the scenario approach, we have found in our previous work that results obtained in scenario studies were very consistent with results obtained in richer yet less controlled settings. We are therefore confident in the validity of the current findings. Nonetheless, we see value in validating and extending the current findings using more ecologically valid procedures, and in the revised paper we explicitly call for future research using such procedures (see p. 22).

References (not mentioned in the paper):

Dai, Z., Galeotti, F., & Villeval, M. C. (2018). Cheating in the lab predicts fraud in the field: An experiment in public transportation. *Management Science*, *64*(3), 1081-1100.

Stamkou, E., Van Kleef, G. A., & Homan, A. C. (2018). The art of influence: When and why deviant artists gain impact. *Journal of Personality and Social Psychology, 115*, 276-303.

4. in their motivational examples, the authors always use firm contexts (interrupting colleagues etc.) - why do they then choose railway examples for the study?

**Response:** We have used the railway scenario because all study participants can easily imagine this setting and most likely have experience with the described situation. In our previous work (see references comment 3), we have used organizational, educational, artistic, and personal settings to study responses to norm violators and found that effects were consistent across settings.

5. my main issue is actually with the signaling idea. If norm violation signals power through being a potentially costly volitional behavior, sanctioning should not necessarily reduce perceived power. The study actually cannot scrutinize this link, as it is a one-shot behavior that is being described. If there were no sanctions, it wouldn´t be costly signaling. Thus, the described mechanism could only work if norm violators keep violating even though there is a chance of being sanctioned - which implies, that in some cases they will be fined, in others not. The design is as it is, but I would like to see a more thorough discussion of this.

**Response:** There is considerable evidence that individuals who violate norms are perceived by others as powerful (for a recent review, see Stamkou, Homan, & Van Kleef, 2020). The theoretical rationale underlying this prediction is that people who violate norms signal that they experience the leeway to act as they please despite normative constraints (Van Kleef et al., 2011). This is a freedom that typically comes with higher rank (Galinsky, Magee, Gruenfeld, Whitson, & Liljenquist, 2008). Accordingly, research has shown that people who violate norms are perceived as having high power (e.g., Van Kleef et al., 2011), status (e.g., Bellezza et al., 2014), and influence (e.g., Stamkou et al., 2018). Regarding the used scenario, individuals who do not buy a train ticket run a (high) risk of being sanctioned. Therefore, by not buying a ticket they signal to be oblivious to normative constraints and can behave as they wish (volition typically reserved for the powerful), which elicit power perceptions in observers. Our study shows that sanctions attenuated the signal of power that norm violator’s apparent volition sends. Observers likely conclude that the sanctioned norm violator does not have the power he seemed to have. In future studies, it would indeed be interesting to investigate how observers will respond to norm violators who keep violating even after having been sanctioned. We added this suggestion to the general discussion section.

References (not mentioned in the paper):

Stamkou, E., Homan, A. C., & Van Kleef, G. A. (2020). Climbing the ladder or falling from grace? A threat-opportunity framework of the effects of norm violations on social rank. *Current Opinion in Psychology, 33*, 74-79.

Galinsky, A. D., Magee, J. C., Gruenfeld, D. H, Whitson, J. A., & Liljenquist, K. A. (2008). Power reduces the press of the situation: Implications for creativity, conformity, and dissonance. *Journal of Personality and Social Psychology, 95*, 1450–1466.

**Responses to Reviewer #2:**

The study deals with an interesting and important topic related to social norms. The methods sound appropriate to test the hypotheses.

I now focus on issues that would help to improve this manuscript:

**Response:** Thank you for this positive comment to our work and your suggestions for improvement.

1. One major issue with this paper is the need to explicate social norms. The literature has been well-documented with norms being conceptualized as injunctive norms and descriptive norms. The association between norms and social sanction has been extensively discussed in the work of Cialdini et al. (1990), Fishbein and Ajzen (2011), and Lapinski and Rimal (2005). Injunctive norms refer to what ought to be done while descriptive norms pertaining to the prevalence of a behavior. Thus, this study seems to intend to deal with injunctive norms rather than descriptive norms. Further, social norms and law are distinct concept (see Rimal & Lapinski, 2015). This study does not seem to distinguish law violation from norm violation. I would think this paper focuses on law and legal sanction, rather a norm-based approach.

**Response:** Although there is a clear conceptual distinction between descriptive and injunctive norms, many common norm violations fall in both categories (Van Kleef, Gelfand, & Jetten, 2019). This is because behaviors that are endorsed as appropriate by the majority of the members of a group (injunctive norms) also tend to be enacted by the majority of the members of a group (descriptive norms). Accordingly, almost all prior research on responses to norm violators has examined behaviors that would be considered violations of both descriptive and injunctive norms. For instance, studies examined responses to individuals who would come in late for a work meeting (Stamkou et al., 2019), put their feet on someone else’s table (Van Kleef et al., 2011), take someone else’s coffee (Van Kleef et al., 2012), park their bike in an illegal spot (Stamkou et al., 2016), or dress improperly for a (professional) occasion (Bellezza, Gino, & Keinan, 2014; Oostrom, Ronay, & Van Kleef, 2021). These are all behaviors that simultaneously infringe on injunctive norms (most people disapprove of these behaviors) and descriptive norms (most people do not exhibit these behaviors). The same is true for the norm violation examined in the current work: Most people believe it is appropriate to buy a train ticket (injunctive norm) and most people indeed do so (descriptive norm). The current operationalization thus reflects the natural conflation of descriptive and injunctive norms in real life. That said, it would be interesting to investigate in future research whether the moderating effect of sanctioning also applies to “pure” violations of descriptive versus injunctive norms. We now refer to descriptive and injunctive norms in the introduction section and we have added this suggestion for future research in the general discussion section.

The reviewer is correct in noting that we did not draw an explicit distinction between social and legal violations. Conceptually, we see legal violations as a subset of the broader category of norm violations. That is, people may violate norms in ways that are or are not punishable, depending on the nature of the infringement and the broader context (e.g., a national law system). In our previous work, we have seen that legal norm violations such as financial fraud (Van Kleef et al., 2011) or illegal parking (Stamkou et al., 2016) elicit similar responses from observers as non-legal norm violations such as arriving late to a meeting (Stamkou et al., 2019) or putting one’s feet on another’s table (Van Kleef et al., 2011): The recurring pattern across these and various other behaviors is that norm violators are perceived by others as powerful. In the revised introduction, we have made it explicit that our focus in the current research was on legal norm violations. Additionally, in the general discussion section (limitations and future directions) we note that previous research indicates that legal and non-legal norm violations elicit similar social responses (p. 23).

References (not mentioned in the paper):

Oostrom, J. K., Ronay, R., & Van Kleef, G. A. (2021). The signalling effects of nonconforming dress style in personnel selection contexts: Do applicants’ qualifications matter? *European Journal of Work and Organizational Psychology, 30,* 70-82.

2. Similarly, the explication of the power concept is limited. Authors define power as the perceived potential to influence others, which is not real power (individuals might not actually hold that power, but only are perceived by others). The lack of explication makes the conceptualization and operationalization of this variable sound less convincing. When we read/see a person not buying a bus ticket, there is little ground to argue that others would think the violator has a great deal of power. The authors use an example of people violating the talking norms in meetings to illustrate their point, but these two contexts are fundamentally different: Some people can talk freely in meetings because they either have real power or the behavior could actually be part of the organizational norms (the meeting norm is that you can interrupt others' talk if you do have something important to say). I would not think a traveler who did not conform to the law as having "a great deal of power," not mentioning that they told lies to authorities. I would think that someone escaping a law sanction as a lucky individual and that should be inferred as the person having power, unless he/she has further actions (ex: making a phone call to powerful others, which is a form of reference power). Such definition and operationalization as written in the paper, therefore, do not sound convincing to me.

**Response:** There is considerable evidence that individuals who violate norms are perceived by others as powerful (for a recent review, see Stamkou, Homan, & Van Kleef, 2020). The theoretical rationale underlying this prediction is that people who violate norms signal that they experience the leeway to act as they please despite normative constraints (Van Kleef et al., 2011). This is a freedom that typically comes with higher rank (Galinsky, Magee, Gruenfeld, Whitson, & Liljenquist, 2008). Accordingly, research has shown that people who violate norms are perceived as having high power (e.g., Van Kleef et al., 2011), status (e.g., Bellezza et al., 2014), and influence (e.g., Stamkou et al., 2018). Furthermore, there is evidence that these perceptions can, under particular circumstances, fuel actual granting of power, for instance via the conferral of control over outcomes, voting, and leadership endorsement (Stamkou et al., 2016; Van Kleef et al., 2012). Our two studies have shown that even individuals who did not buy a train ticket were perceived as more powerful. To acknowledge that our research speaks to *perceived* power, we have made this explicit throughout the paper (e.g., p. 4, pp. 9-13, pp. 15-17, p. 20, p. 22).

References (not mentioned in the paper):

Stamkou, E., Homan, A. C., & Van Kleef, G. A. (2020). Climbing the ladder or falling from grace? A threat-opportunity framework of the effects of norm violations on social rank. *Current Opinion in Psychology, 33*, 74-79.

Galinsky, A. D., Magee, J. C., Gruenfeld, D. H, Whitson, J. A., & Liljenquist, K. A. (2008). Power reduces the press of the situation: Implications for creativity, conformity, and dissonance. *Journal of Personality and Social Psychology, 95*, 1450–1466.

3. I see that it is quite controversial to argue that freedom to do something would always lead to inferences of power possession. It might only signal power as the authors suggest under some certain conditions (there should be boundary conditions). I would think that people can think of someone who acts as she/he wants, which deviates from social approval, as having less power. This rival theory can be illustrated, for example, by historical accounts related to social movements in which less powerful individuals in a society (both real and perceived) violate a political norm/law to gain power. We may also see drivers overspeed and think of them as traffic violators who would likely confront more powerful others (policemen). This social comparison will likely lead to perceptions of the violators as being less powerful, or even having no power and thus defying law to satisfy their desired power. In the same vein, a person did not buy a ticket might mean he/she has no other choice (lack of freedom) and thus violates the law (no power). This line of reasoning shows that the theorization of the model in this paper seems problematic because the authors left too many rival theories unaddressed.

**Response:** Please, see our response to your comment 2 and the recent review of Stamkou et al., 2020. In our paper we recognize that potentially costly behavior (e.g., a norm violation) can only act as a signal of an underlying trait (e.g., power) in the absence of additional cues that provide direct information about that trait (see p. 4). Hence, norm violation may not signal power to the same degree when additional information is available. Yet, if additional information is lacking (which seems also the case in your example of the speedy driver), bystanders tend to ascribe power to the norm violator as has been repeatedly demonstrated in prior research and in the current study. We stress this in the discussion section on pp. 21-22. To our best knowledge, our study is one of the few examining a boundary condition, namely sanctioning. Future research could investigate other boundary conditions, such as the background of the norm violator (which can act as an additional information cue). For instance, if people are aware of the power of the person violating the norm – a situation that you raise in your comment above – this should act as a moderator of the effect. Distantly speaking to your comment, we have some unpublished research that suggests that outgroup members breaking the norm are not seen as more powerful, whereas ingroup members who break the norm are seen as more powerful. We have added this suggestion to the discussion section (p. 22).

4. When theorization is not sound, having supporting data does not help much. The three key variables likely often have some sorts of correlations. A statistical model can be statistically significant without any theoretical background. Plus, the idea that someone has power could have more freedom to do things, even violating a law is not new in the literature. Also, the idea of sanctioning someone reducing his/her power offers no novel theoretical implication (someone goes to jail of course will normally have much less power than before). I do not see how such theorizations add to the literature.

**Response:** The key contribution of our study is not that sanctioning reduces power per se, but that sanctioning reduces the effect of norm violation on power perceptions. Our work is based on costly signaling theory (Bergmüller et al., 2007; Zahavi, 1995) and prior empirical evidence for the norm violation 🡪 volition 🡪 power chain (see Stamkou et al., 2020). A robust finding (in individualistic societies) is that norm violators are perceived by others as powerful and high status. This is important, because people often defer to (and are less likely to speak up to) others whom they perceive as powerful (Anderson & Kilduff, 2009; Cheng, Tracy, Foulsham, Kingstone, & Henrich 2013). Moreover, norm violators are sometimes granted power and leadership due to the impression they make on others, and this increased power in turn makes future violations more likely (Van Kleef, Wanders, Stamkou, & Homan, 2015). To the degree that this vicious cycle is a cause for concern, it is important to understand how it can be broken. The current research provides first evidence that sanctioning can sever the link between norm violation and power perceptions, thereby disrupting a potentially toxic spiral of norm violation and power abuse. Future research can build on our work and provide further evidence for the role of sanctions or other punishing responses (e.g., informal (social) punishment) in preventing people from gaining influence through norm violations.

References (not mentioned in the paper):

Cheng JT, Tracy JL, Foulsham T, Kingstone A, Henrich J. Two ways to the top: evidence that dominance and prestige are distinct yet viable avenues to social rank and influence. J Pers. Soc. Psychol. 2013; 104(1). 103–125. doi: 10.1037/a0030398.

That says, I commend the authors on engaging in a project with a rigorously methodological design. I sincerely appreciate the author(s)’ work, and I wish them the best of luck with this project.

**Response:** We appreciate your challenging and constructive comments.

**Responses to Reviewer #3:**

This manuscript reports two experiments designed to test the hypothesis that norm violators will appear less powerful when they are punished than when they are not. The experiments build on earlier research showing that norm violators are perceived as more powerful than norm abiders; they introduce sanctions as a moderator of this effect.

The manuscript has a number of strengths: The research is methodologically sound; the analyses are appropriate; the write-up is clear and complete. At the same time, the research makes a very modest contribution to the literature, even more modest than the write-up suggests. It mainly shows that an effect previously demonstrated by these investigators has limited scope. It is good to know that, of course, but it does not represent the level of contribution typically found in PLOS ONE articles.

**Response:** Thank you for your positive but also critical general comment.

Let me describe briefly how I would interpret the results of this research, as my interpretation is somewhat different from how the authors frame the results. These results demonstrate that in a situation in which there are rules for how to behave, people are sensitive to where the power lies: with the rules or with the individuals. (I’m using rules here, rather than norms, because the research scenario conflates the two, but the same analysis holds for norms.) To the extent that people follow the rules and violations of the rules are enforced, power lies with the rules; to the extent that people violate the rules and get away with it, power lies with the individuals. Volition, on the other hand, depends on whether people try to follow the rules. Study 1 shows that neither rule-abiders nor rule-violators have much power if the rules are enforced, but if the rules are not enforced, even people who accidentally violate them (by not having their ticket to present to the conductor) have power. Study 2 shows that rules are powerful when people abide by them and when they are enforced; the relationship between individual volition and power is strongest when rules are weak. This summary captures all of the findings of this research and is entirely consistent with current views of how social rules and norms work. They clarify that the earlier finding of greater power attributed to norm violators holds only when norms are weak, but that simply serves to limit the scope and importance of the earlier finding. It does not challenge or extend current understandings of the way norms work.

I will leave to the editor the decision of whether this manuscript makes enough of a contribution to warrant publication in PLOS ONE. Regardless of where it is published, I think some revision is in order to simplify and clarify the presentation and interpretation of the results.

**Response:** Our research is indeed built on solid theory and prior empirical evidence. We believe it is valuable to test theory in different context and with different methods. Also, it is good science to examine the boundary conditions of a theory. In this study, we do both: replicating prior evidence - but now in an experimental context where a legal norm is violated - and testing a boundary condition (sanctioning). Indeed, we show that norm violators gain more power in the eyes of bystanders when norms are not enforced by sanctions, which we believe is a highly relevant finding for dealing with norm violating behaviors in society. This finding may also hold for the violation of non-legal norms where the sanction is not a fine established by law or official rule but rather depends on the responses of bystanders. We plan further research into the ‘self-reinforcing loop’, by which norm violators appear powerful, bystanders submit to and consolidate the power of the norm violators and thus encourage further norm violation, can be broken (see p. 22). Based on your comment and the comments of the other reviewers we revised the text of the paper to clarify our conceptualizations and the interpretation of the results (see pp. 5, 21-24).

**Responses to Reviewer #4:**

Summary of the paper

The authors (1) replicate previous research that third-party observers believe that norm violators have a greater volition and power than norm abiders, and (2) extend that research to understand whether sanctions can be used to reduce perceptions of power associated with norm violation. The authors conduct two studies: (1) with a German online sample and a 2X2 design (Abiding norm, violating norm)X(Sanctions, No Sanctions) and (2) with a Dutch online sample with 1X3 design (Abiding Norm), (Violating Norm X Sanctions), (Violating Norm X No Sanctions).

The authors use a vignette about a passenger buying or not buying a ticket on a train, and a controller either sanctioning (or not) the passenger who fails to show a ticket. They measure survey respondents’ perceptions of the passenger’s volition and power using survey questions. The authors claim that the main mechanism of how norm violation affects power is through volition i.e., a passenger who violates a norm is considered to act on their own volition, and this belief about volition leads to increased perceptions about the power they possess. The authors find that sanctions reduce the perceptions of power of the passenger irrespective of whether they are norm abiding or not, and irrespective of their volition (Result of Study 1). They find weak evidence for the mechanism that norm violation affects power through volition.

The paper is well-written, and the data collection and analysis are well-done.

**Response:** Thank you for your positive assessment of our work and your detailed comments.

Major critique

1. The paper clearly shows that introduction of sanctions reduces power associated with both norm-abiding and norm-violating individuals. However, the mechanism that norm violation leads to increased volition that further leads to increased power is not supported by evidence. The authors cannot claim that the mechanism is true unless they vary volition exogenously and find that perceptions of power are affected by that variation.

**Response:** There is ample evidence from previous work that individuals who violate norms are perceived by others as having high volitional capacity, which in turn fuels perceptions of power and influence (e.g., Bellezza et al., 2014; Stamkou et al., 2018; Van Kleef et al., 2011). We replicate this link between volition and power. However, we agree that we didn’t manipulate volition and thus cannot claim causal evidence for this link. We address this point in the limitations and future directions section of the manuscript (see p. 22).

2. The results from Study 1 suggest that introduction of sanctions reduce perceptions of passenger’s power irrespective of his/her volition and his/her norm abidance/violation. In study 2, the authors find a different result because that they do not have a treatment with sanctions for norm-abiding behavior in this Study and thus do not have much variation in volition. I don’t think we can conclude from Study 2 that the claimed mechanism (Norm Violation-->Increased volition-->Increased power) is true.

**Response:** The issue of sanctions for norm-abiding behavior is something we discussed at length in the author team. On the one hand, from the point of view of having orthogonal manipulations and allowing different comparisons between conditions, it is indeed desirable to include a condition in which norm-abiding behavior is sanctioned. On the other hand, from the point of view of validity, including such a condition is not desirable as it undermines the credibility of the scenario and makes the interpretation of comparisons with that condition less straightforward. We solved this dilemma by running two studies, one of each type, so that the disadvantages of one approach are remedied by the advantages of the other approach. We believe that in conjunction the two studies provide reasonable support for the presumed theoretical mechanism of volitional capacity. We have made these considerations more explicit in the revision (introduction Study 2 and limitations in the general discussion section; p. 13 and 21 respectively).

3. Moreover, volition and power are correlated. However, there is no evidence that it is higher volition that leads to greater power. It could be the other way round where higher power leads to having greater volition.

**Response:** Please, see our response to your comment 1.

4. It is not clear what “power” means in the context of a passenger who either buys or does not buy a ticket on a train. How does not buying a ticket make one more influential? A better way to measure power in this situation would be to see if the third-party observer is more likely to follow instructions from someone who violated the norm versus who obeyed the norm.

**Response:** There is considerable evidence that individuals who violate norms are perceived by others as powerful (for a recent review, see Stamkou, Homan, & Van Kleef, 2020). The theoretical rationale underlying this prediction is that people who violate norms signal that they experience the leeway to act as they please despite normative constraints (Van Kleef et al., 2011). This is a freedom that typically comes with higher rank (Galinsky, Magee, Gruenfeld, Whitson, & Liljenquist, 2008). Accordingly, research has shown that people who violate norms are perceived as having high power (e.g., Van Kleef et al., 2011), status (e.g., Bellezza et al., 2014), and influence (e.g., Stamkou et al., 2018). Furthermore, there is evidence that these perceptions can, under particular circumstances, fuel actual granting of power, for instance via the conferral of control over outcomes, voting, and leadership endorsement (Stamkou et al., 2016; Van Kleef et al., 2012). Replicating previous studies, we also found that norm violation - operationalized as a passenger who did not buy a ticket on a train - elicited perceptions of power. Since people tend to submit to powerful others (DeRue & Ashford, 2010; Epitropaki et al., 2013; Van Kleef et al., 2012) it would indeed be valuable to investigate - in a similar context as in the current study - if observers are also more likely to follow instructions from norm violators (p. 22). Thank you for this interesting suggestion. We have added it to the general discussion section.

5. The payment to participants is small and probabilistic. For example, the participants had a 20% chance of winning a 10Euro voucher in Study 2. It is unclear how seriously the participants took the survey with these small incentives.

**Response:** We are confident that our findings are based on data of participants who took the survey seriously. First, we excluded participants who did not finish the questionnaire and therefore did not invest enough effort. Second, we excluded participants who failed the attention checks. Third, the manipulation checks showed that the intended differences between conditions in the presence or absence of norm violation and sanctions were achieved. Finally, although we believe that all participants who completed the questionnaire were sufficiently motivated, participants were randomly assigned to conditions and thus we may assume that participants’ motivation is the same across conditions.

6. The authors use a vignette about the norm of buying a ticket or not on a train. The authors may want to discuss how this specific situation can be generalized to other situations.

**Response:** Previous work has revealed that very different norm violations across a variety of settings have very similar effects on perceptions of power. For instance, studies examined responses to individuals who would come in late for a work meeting (Stamkou et al., 2019), put their feet on someone else’s table (Van Kleef et al., 2011), take someone else’s coffee (Van Kleef et al., 2012), park their bike in an illegal spot (Stamkou et al., 2016), or dress improperly for a (professional) occasion (Bellezza, Gino, & Keinan, 2014; Oostrom, Ronay, & Van Kleef, 2021). These are all behaviors that people typically neither approve nor exhibit. The same is true for the norm violation examined in the current work: Most people find it appropriate to buy a train ticket and they behave accordingly. All in all, we believe that the findings reported in the current paper are likely to generalize to other types of norm violations and other settings.

References (not mentioned in the paper):

Oostrom, J. K., Ronay, R., & Van Kleef, G. A. (2021). The signalling effects of nonconforming dress style in personnel selection contexts: Do applicants’ qualifications matter? *European Journal of Work and Organizational Psychology, 30,* 70-82.

Minor critique

1. When you say norms, can you clarify if they are descriptive or prescriptive norms?

**Response:** Although there is a clear conceptual distinction between descriptive and prescriptive (injunctive) norms, many common norm violations fall in both categories (Van Kleef, Gelfand, & Jetten, 2019). This is because behaviors that are endorsed as appropriate by the majority of the members of a group also tend to be enacted by the majority of the members of a group. Accordingly, almost all prior research on responses to norm violators has examined behaviors that would be considered violations of both descriptive and injunctive norms (see Bellezza et al., 2014; Oostrom et al., 2021; Stamkou et al., 2016; Stamkou et al., 2019; Van Kleef et al., 2011; Van Kleef et al., 2012). The same is true for the norm violation examined in the current work: Most people believe it is appropriate to buy a train ticket and most people indeed do so. The current operationalization thus reflects the natural conflation of descriptive and injunctive norms in real life. We now refer to descriptive and injunctive norms in the introduction section of the revised paper.

2. Both the sanction and no sanction conditions in the paper technically have sanctions, in one case they are enforced and in another they are not. The authors can clarify that by changing their terminology.

**Response:** We agree that not buying a train ticket carries the risk of a formal penalty that may be enforced or not. The sanctioning in our paper refers to a formal (legal) rather than an informal (social) punishment. Depending on the experimental condition in Study 1, a sanction was imposed or not when the violator exhibited sanctionable behavior, that is, could not show a ticket to the controller (see page 14). In the overview of our study (page 5) we now state that sanctions refer to formal sanctions.

3. 75% of the sample is women and is not representative of the German population in Study 1. The authors may want to discuss how the gender composition of their sample may affect the result.

**Response:** We agree that the gender composition in Study 1 is not representative for the German population. However, we have no specific assumptions about possible gender differences in individual responses to the violation of legal norms. Moreover, participants were randomly assigned to conditions and the findings of the two studies are largely the same and in line with previous research. We now discuss the gender composition of our studies in the general discussion section (p. 23).

4. Since Study 1 and 2 are conducted with different populations (German vs Dutch online samples), the others should comment on how comparable these studies are. Are there differences in norms of ticket buying in these two populations?

**Response:** There are cultural differences as well as similarities between the two countries in dimensions that are relevant to norm violation. Germany scores higher than the Netherlands on cultural tightness, which relates to the importance that is attached to rules and the severity of punishment for violations (Gelfand et al., 2011). In Study 1 (German sample) we found that non-sanctioned norm abiders were perceived to have violated norms to a greater extent than sanctioned norm abiders, although the effect size was small. We could speculate that participants were aware of the railway operators’ right to fine the traveler and – due to their tightness - were indignant that the traveler got away without this fine.

Germany and the Netherlands are comparable in terms of individualism, the degree to which uniqueness, personal achievement, and self-expression are valued (as opposed to group harmony and collective outcomes). A cross-cultural comparison of responses to norm violations (including data from Germany and the Netherlands) revealed that particularly respondents from individualistic cultures (including Germany and the Netherlands) perceive norm violators as more powerful than norm abiders (Stamkou et al., 2019).

Finally, regarding the specific scenario in this study, we would like to note that Germany and the Netherlands are European Schengen states, allowing free traveling among these states, and therefore use similar terms and conditions for train travel. In the discussion section (p. 24), we now address the possibility of cultural differences as a subject for future research.
