# Supplementary material for: How norm violators rise and fall in the eyes of others: The role of sanctions
Source: PLoS One. 2021 Jul 29;16(7):e0254574. doi: 10.1371/journal.pone.0254574 (PMC8324048; doi:10.1371/journal.pone.0254574)
Supplement: Attachment — Submitted filename: Response to reviewers R2 .docx [file pone.0254574.s003.docx]

**Responses to Editor comments**

Thank you for submitting your manuscript to PLOS ONE. After careful consideration, we feel that it has merit but does not fully meet PLOS ONE’s publication criteria as it currently stands. Therefore, we invite you to submit a revised version of the manuscript that addresses the points raised during the review process.

**Response:** Thank you for giving us the opportunity to submit a second revised version of our manuscript.

In the revised version of the paper, please try to clarify the aspects related to descriptive and injunctive norms, how the perceived power has been measured, the specificity / limitations of the study. When revising the paper, please consider the reviewers' comments listed at the bottom of the email.

**Response:** We have carefully considered the comments of Reviewer 2 and clarified the concepts of descriptive and injunctive norms, the measurement of perceived power, and the limitations of the study.

**Responses to Reviewer #2:**

I appreciate the authors’ efforts to address reviewers’ comments.

**Response:** Thank you for your additional and helpful comments.

1. Descriptive and injunctive norms were not clearly defined in the revised manuscript. These norms are individually perceived. Social norms can be examined at the collective level, which is different from social norm existing at the individual/perceived level. At the collective level, both types of norms can converge, but not necessarily so at the perceived level. People are not always cognizant of the prevailing descriptive or injunctive norms in certain contexts (please see Tankard & Paluck, 2016). The social norm approach, therefore, suggests that misperception of social norms is a problematic issue for norm-violating behaviors (please see Berkowitz, 2005).

**Response:** Thank you for your concrete suggestion for better defining descriptive and injunctive norms. In the revised manuscript, we now state on page 3: *“Injunctive and descriptive norms are individually perceived but when people are cognizant of prevailing norms and endorse these norms, both types of norms can converge and be shared at the collective level [18]”*.
Also, please note that in our studies, people did perceive the behavior of the norm violator to be violating of norms (which is evident from the strong main effects on the manipulation check; eta squared = .845 in S1, and eta squared = .715 in S2). So, even though not everyone is always aware of the prevailing (descriptive or injunctive) norms in a certain situation, the behavior we studied in our research seems to be perceived consistently across participants.

The authors wrote that injunctive norms and descriptive norms almost always work in the same directions. They wrote that behaviors that are endorsed as appropriate by the majority of the members of a group (injunctive norms) also tend to be enacted by the majority of the members of a group (descriptive norms). However, there are many situations where these two types of normative influences do not overlap, such as when people approve of, but do not practice, particular behaviors (Cialdini et al., 1990). Descriptive norms and injunctive norms can also be antagonistic, and they may provide us with conflicting information about normative behaviors in a given context (Lapinski & Rimal, 2005). For example, consider these norm-violating behaviors: drinking, smoking, speeding, etc. (please see, for example, Chung & Rimal, 2016; Hue et al., 2015).

The authors responded that “almost all prior research on responses to norm violators has examined behaviors that would be considered violations of both descriptive and injunctive norms.” Perhaps, this manuscript needs to speak for itself as to why these two types of norms are almost all considered in such a way? Also, it might be necessary to address other theoretical frameworks that argue otherwise. For instance, the focus theory of normative conduct (Cialdini et al., 1990), the theory of normative social behavior (Lapinski & Rimal, 2005), the reasoned action approach (Fishbein, 2009) suggest that violation of injunctive norms does not necessarily go along with violation of descriptive norms, and vice versa.

**Response:** We fully agree that there are situations in which descriptive and injunctive norms do not overlap and that violation of injunctive norms does not necessarily go along with violation of descriptive norms. Your comment led us to realize that the claims we made about the frequent convergence of descriptive and injunctive norms we provided in the previous version of the paper may have been too strong, and we have therefore moderated our claims in the new revision. That said, in our studies we explicitly used a scenario in which a formal norm (a contract between the company operating the train and the passenger using its services) is violated (not buying a train ticket) that is likely endorsed and enacted by most members of a (western) society. In the revision, we have connected our claims about convergence of descriptive and injunctive norms more tightly to this specific operationalization so as not to imply that such convergence always occurs. To keep a clear focus in our paper, we decided not to elaborate further on other theoretical frameworks that address conflicting information about normative behaviors and possible discrepancies between the violation of injunctive and descriptive norms. Instead, in our discussion section we now reflect on our assumption that the participants in our study were cognizant of the norm to buy a train ticket and tended to endorse and enact this norm. The added information reads (page 24: *“Third, we considered the norm violation in this study as a violation of a descriptive and injunctive legal norm.* *We assumed that buying a train ticket is a well-known legal norm enacted and endorsed as appropriate by most study participants. Although we did not test this assumption, the results of the manipulation checks in both studies showed that participants perceived the behavior of the norm violator to be violating of norms”* and “*Future research on norm violation could pay more attention to the actual endorsement and enactment of specific norms among study participants. Additionally, future research could examine situations where the violation of an injunctive norm does not constitute a violation of a descriptive norm and vice versa [43-44] to understand how participants differentially respond to violations of such more complicated normative influences”.*

The authors commented that they see legal violations as a subset of the broader category of norm violations. So, it looks like this research approach suggests that violating the law also means violating social norms. To this point, please address this argument from social norm theorists:

“Different from laws, norms are socially negotiated and contextually dependent modes of conduct; laws are explicitly codified proscriptions that link violations with their corresponding punitive measures. Laws are not socially negotiated (although their enforcement might be), whereas norms and their transgressions, by definition, are negotiated through social interaction. This is an important criterion because it explains why the same mode of conduct (e.g., littering) is acceptable in one social context (littered environment) but not in another (clean environment; Cialdini, Reno, & Kallgren, 1990). Laws and norms can certainly reinforce each other. For example, smokers may choose to refrain from lighting up in a public place for a number of reasons, including legal (fear of being penalized) or normative (fear of being accosted by someone in the vicinity), both of which lead to the same outcome (not lighting up). At other times, the two may act in opposition to each other, as when underage college students follow alcohol-drinking norms despite this behavior being illegal.” (page 394, Rimal & Lapinski, 2015)

**Response:** Again, thank you for your concrete input. We now address the difference between laws and norms in the Discussion section. Our text on page 24 reads as follows: *“**Train passengers who do not buy a train ticket transgress a legal norm and run the risk of being formally penalized by means of a fine. Note that laws, as opposed to social norms, are not negotiated through social interaction, which means that people’s responses to violating the legal norm to buy a train ticket may be relatively similar across social contexts [42]. Prior research has shown that legal norm violations such as financial fraud [5] or illegal parking [23] elicit similar responses from observers as non-legal norm violations such as arriving late to a meeting [8] or putting one’s feet on another’s table [5]”.*

2. This study focuses on the association between norm violation and perception of power. The authors defined power as the perceived potential to influence others. Additionally, they suggested that the perception of someone having the capacity to do what that someone wants, it signals that the person has the capacity to influence others (perception of power; line 58-60, page 4). Following this logic, a person who does not buy a ticket is perceived as having the potential to influence others. I am still confused with this logic. How is it possible that we travel on a bus and witness a stranger not buying a ticket would make us think that that person has the potential to influence us and others? In this scenario, I might think that the person possesses some degrees of autonomy to conduct such a behavior. Yet, autonomy is conceptually different from power and does not always lead to the attribution of power. So, an inference from a high degree of autonomy to a high level of power sounds like a leap in logic. The authors cited several studies to back up this argument in their response, but the manuscript should speak for itself considering that this is a pivotal theorization in this study.

Additionally, the authors wrote that “people who violate norms demonstrate that they can behave as they wish.” How do we tell if people would attribute someone who does not buy a ticket either as the person wishes to do so or that the person has no choice at all? If the attribution is related to the second scenario, does that still mean that the person is perceived to have the capacity to influence others? This situation seems to relate to observers’ perceptions of efficacy of a norm violator as well as observers’ attributions of the norm violator's traits. Attribution theory suggests that human tends to attribute others’ negative behaviors as causally due to internal factors and with less positive traits (e.g., fundamental attribution errors). As such, a norm violator can be attributed with more negative attributes (e.g., poor, desperate) than positive attributes (e.g., rich, high self-efficacy). Isn’t it logical to think that positive attributes would be more likely to associate with higher perception of power?

**Response:** In order to better explain the link between norm violation and perceptions of power, we have revised the text on pages 3 and 4. The text on page 3 now reads as follows: *“**Social norms – implicit or explicit rules or principles that are understood by members of a group and that guide and/or constrain behavior [1] – create a shared understanding of what is acceptable within a given context and thereby contribute to the functioning of social collectives [2-4]. Accordingly, research has documented that people who violate norms tend to elicit negative responses in others, including unfavorable social perceptions [5], negative emotions [6- 8], scolding [9], gossip [10], and punishment [11-13]. Intriguingly, however, research has also demonstrated that norm violators are perceived as powerful [5], high in status [14], and influential [15]”.*

The text on pages 3-5 now reads as: *“**By ignoring the norms that bind others, norm violators demonstrate that they can act as they wish and do not fear interference from others [5]. This is a freedom that typically comes with higher rank [19]. The influential approach/inhibition theory of power [20] states that power, which is commonly defined as asymmetrical control over valuable resources that enables influence, liberates behavior, whereas powerlessness constrains it. Indeed, ample research supports that power renders people more likely to act, even if the resulting behavior is inappropriate or harmful [21-22]. Because behavioral freedom is thus intimately associated with power, people who observe unchecked behavior of others may make inferences about others’ level of power. Indeed, people who act as they wish and disregard social norms are perceived as having high status [14], influence [15], and power [5]. Furthermore, these perceptions can, under particular circumstances, fuel actual granting of power, for instance via the conferral of control over outcomes, voting, and leadership endorsement [23-24]. In line with the notion that power liberates behavior, previous research has demonstrated that norm violators are perceived as powerful because they appear to experience the freedom to act as they please [14-15, 5] – that is, they are high on volitional capacity. In other words, norm violators are perceived as powerful because their behavior signals an underlying quality, namely the freedom to act at will. This argument resonates with costly signaling theory [25-26]**, which states that any seemingly costly behavior (involving large investments or risks of receiving negative outcomes) functions as a signal of an underlying characteristic [25-26].* *An example of costly behavior is the reckless driving of young men as to show their strength and skills to peers and potential mates, risking serious injury or death* *– a type of behavior that is under particular circumstances “rewarded” with power [27]. Norm violations are potentially costly as they are frequently sanctioned [14] by means of formal (e.g., legal) punishment [28] and/or informal (social) punishment (e.g., anger, social exclusion [29-30]). According to costly signaling theory, people who engage in potentially costly norm-violating behavior signal that they possess traits that allow them not to worry about interferences from others.* *Because this capacity to do what one wants is typically reserved for the powerful [31], norm violators appear powerful* *when there are no additional cues that provide direct information about this attribute [5]”.*

Thank you for your thoughts about other (than power) perceptions of norm violators. Your intuition that norm violators are generally perceived negatively is borne out by previous research, which we believe makes it all the more interesting that people still perceive norm violators as powerful – except when they are sanctioned, as we demonstrate in the current paper, because sanctioning severs the link between perceived volitional capacity and perceived power. In the introduction section on page 3, we now briefly discuss previous work that has documented negative responses to norm violations to better contextualize the current findings and enable nuanced conclusions. Your comment also led us to think about associations between perceived power and other social perceptions of norm violators more broadly, which we will seriously consider when preparing future research on responses to norm violations.

3. Operationalization of perceived power: It might be helpful to see the specific items used to measure perceived power. Right now, the manuscript says that the authors measured this construct by items like “I think this person has a great deal of power,” which does not tell if participants understood that power was about the potential ability to influence themselves and others. It would also be more informative for reviewers and readers to see the specific items measuring other scales because the items were adapted to this research situation.

**Response:** In the method section of Study 1, we refer to the supplementary material containing the scale items.

Perceived Power (Anderson & Galinsky, 2006) was measured with the following items:

1. He can get people to listen to what he says.

2. His wishes do not carry much weight. [reverse scored]

3. He can get others to do what he wants.

4. Even if he voices them, his views have little sway. [reverse scored]

5. He thinks he has a great deal of power.

6. His ideas and opinions are often ignored. [reverse scored]

7. Even when he tries, he is not able to get his way. [reverse scored]

8. If he wants to, he gets to make the decisions.

In addition, we have added one more sample item of the perceived power scale in the Method section of Study 1.

4. What has been the common context of the studies the authors cited? Were these studies mostly conducted in the western context where law and order and transportation infrastructure are to some extent more stable than that in developing countries? It is hard to fathom that a thieve on a public bus in a non-western country (norm violator) would be perceived by on-lookers as having the potential to influence others (power). It is also hard to think of an illegal drug user as being someone who has power to influence others. I wonder if there is such a line of research related to this study’s main theoretical framework to be able to be generalized with a global implication. Even in the review of Stamkou et al. (2021) that the authors cited, this norm violation – perceived power linkage was shown to have contradicting effects in India. To this point, I still see that there’s a significant challenge to persuade readers of the causal link between the observation of norm-violation behaviors and perceived power.

**Response:** With regard to the question of whether a thieve on a bus would be perceived as being capable of influencing others, we believe the answer is a clear yes. Interpersonal influence stems not only from admirable qualities such as competence, expertise, and skill (which are related to prestige) but also from attributes such as assertiveness, intimidation, and coercion (which are related to dominance); see, for instance, Anderson and Kilduff (2009) and Cheng et al. (2013). With regard to culture, the studies we cite were mostly conducted in a western context, consequently showing a link between norm violation and observers’ power perceptions. Stamkou et al.’s (2019) cross-cultural comparison of responses to norm violations revealed that the link between norm violation and power perceptions is positive in individualistic cultures, but negative in collectivistic cultures (Stamkou et al., 2019). Moreover, individuals in tighter cultures are less willing to endorse norm violators as leaders, compared to those in looser cultures. It is clear from this cross-cultural study that observers’ responses to norm violations are indeed influenced by the cultural context in which the violation occurs. We have addressed this issue in the final paragraph of the Discussion section on page 25.

5. The citation of perceived norm types should be acknowledged to Cialdini et al. (1990) who coined the terms, which then became widely adopted in social science.

**Response:** Thank you for noting this omission. We have included the reference to Cialdini et al. (1990).

6. The term “costly behavior” should be clearly defined and with an example. Perhaps, not all readers will have the in-depth knowledge of the authors’ research discipline.

**Response:** We have revised the text on costly behavior. The text on page 5 now reads as: *“This argument resonates with costly signaling theory [25-26], which states that any seemingly* *costly behavior (involving large investments or risks of receiving negative outcomes) functions as a signal of an underlying characteristic [25-26]. An example of costly behavior is the reckless driving of young men as to show their strength and skills to peers and potential mates, risking serious injury or death – a type of behavior that is under particular circumstances “rewarded” with power [27].”*

**References not included in the paper:**

Cheng, J. T., Tracy, J. L., Foulsham, T., Kingstone, A., & Henrich, J. (2013). Two ways to the top: Evidence that dominance and prestige are distinct yet viable avenues to social rank and influence. *Journal of Personality and Social Psychology*, *104*, 103-125.

**References added to the paper:**

**1.** Cialdini RB, Trost MR. Social influence: Social norms, conformity, and compliance. In Gilbert DT, Fiske ST, Lindzey G, editors. Handbook of social psychology. New York, NY: McGraw-Hill; 1998. pp. 151–192.

**2.**  Jetten J, Hornsey MJ. Deviance and dissent in groups. Annu Rev Psychol. 2014; 65: 461–485. [doi: 10.1146/annurev-psych-010213-115151](https://doi.org/10.1146/annurev-psych-010213-115151).

**3.**  Tomasello M, Vaish A. Origins of human cooperation and morality. Annu Rev Psychol. 2013; 64: 231–255. doi: 10.1146/annurev-psych-113011-143812.

**6.**  Gutierrez R, Giner-Sorolla R. Anger, disgust, and presumption of harm as reactions to taboo-breaking behaviors. Emotion. 2007; 7(4): 853–868. doi: 10.1037/1528-3542.7.4.853 853.

**7.** Ohbuchi KI, Tamura T, Quigley BM, Tedeschi JT, Madi N, Bond MH, Mummendey A. Anger, blame, and dimensions of perceived norm violations: Culture, gender, and relationships. J Appl Soc Psychol. 2004; 34(8): 1587–1603. doi: 10 .1111/j.1559-1816.2004.tb02788.x.

**9.** Vaish A, Missana M, Tomasello M. Three-year-old children intervene in third-party moral transgressions. Br J Dev Psychol. 2011; 29(1): 124–130. doi: [10.1348/026151010X532888](https://doi.org/10.1348/026151010X532888).

**10.** Beersma B, Van Kleef GA. Why people gossip: An empirical analysis of social motives, antecedents, and consequences. J Appl Soc Psychol. 2012; 42(11): 2640–2670. doi: 10.1111/j.1559-1816.2012.00956.x.

**11.** Fehr E, Fischbacher U. Third-party punishment and social norms. Evol Hum Behav. 2004; 25(2): 63–87. doi: 10.1016/S1090-5138(04)00005-4.

**12.** Marques JM, Abrams D, Serôdio RG. Being better by being right: Subjective group dynamics and derogation of ingroup deviants when generic norms are undermined. J Pers Soc Psychol. 2001; 81(3): 436–447. doi: [10.1037/0022-3514.81.3.436](https://doi.org/10.1037/0022-3514.81.3.436).

**13.** Yamagishi T. The provision of a sanctioning system as a public good. J Pers Soc Psychol. 1986; 51(1): 110–116. doi: [10.1037/0022-3514.51.1.110](https://doi.org/10.1037/0022-3514.51.1.110).

**15.** Stamkou E, Van Kleef GA, Homan AC. The art of influence: When and why deviant artists gain impact. J Pers Soc Psychol. 2018; 115(2): 276-303. [doi: 10.1037/pspi0000131](https://doi.apa.org/doi/10.1037/pspi0000131).

**18.** Tankard ME, Paluck EL. Norm perception as a vehicle for social change. Soc Iss Policy Rev. 2016; 10(1): 181-211. doi: [10.1111/sipr.12022](https://doi.org/10.1111/sipr.12022).

**19.** Galinsky AD, Magee JC, Gruenfeld DH, Whitson JA, Liljenquist KA. Power reduces the press of the situation: Implications for creativity, conformity, and dissonance. J Pers Soc Psychol. 2008; 95(6): 1450–1466. doi: 10.1037/a0012633.

**20.** Keltner D, Gruenfeld DH Anderson C. Power, approach, and inhibition. Psychol Rev, 2003; 110(2): 265-284. doi: 10.1037/0033-295X.110.2.265.

**21.** Cho M, Keltner D. Power, approach, and inhibition: Empirical advances of a theory. Cur Opin Psychol. 2020; 33: 196-200. doi: 10.1016/ j.copsyc.2019.08.013.

**22.** Pike BE, Galinsky AD. Power leads to action because it releases the psychological brakes on action. Cur Opin Psychol. 2020; 33: 91-94. doi: 10.1016/J.COPSYC.2019.06.028.

**27.** Van Kleef GA, Heerdink MW, Cheshin A, Stamkou E, Wanders F, Koning LF, et al. No guts, no glory? How risk-taking shapes dominance, prestige, and leadership endorsement. J Appl Psychol. (in press).

**42.** Rimal RN, Lapinski MK. A re-explication of social norms, ten years later. Commun Theor. 2015; 25(4): 393-409. [doi: 10.1111/comt.12080](https://doi.org/10.1111/comt.12080).

**43.** Cialdini RB, Reno RR, Kallgren CA. A focus theory of normative conduct: Recycling the concept of norms to reduce littering in public places. J Pers Soc Psychol. 1990; 58(6): 1015-1026. doi: 10.1037/0022-3514.58.6.1015.

**44.** Lapinski MK, Rimal RN. An explication of social norms. Commun Theor. 2005; 15(2): 127-147. doi:10.1111/j.1468-2885.2005.tb00329.x
